# Percheron thalamopeduncular syndrome with cervical dystonia: A case report

**DOI:** 10.1590/s1980-5764-2016dn1004019

**Published:** 2016

**Authors:** Luiz Felipe Vasconcellos, Chan Tiel, Felipe Kenji Sudo, Denise Madeira Moreira, Eliasz Engelhardt

**Affiliations:** 1 Institute of Neurology Deolindo Couto - Federal University of Rio de Janeiro (UFRJ), RJ, Brazil.; 2 PROPSAM-Institute of Psychiatry-UFRJ, Rio de Janeiro, RJ, Brazil.; 3 Cognitive and Behavioral Neurology Unit - Institute of Neurology Deolindo Couto - Institute of Psychiatry (CDA/IPUB) - UFRJ, Rio de Janeiro, RJ, Brazil.

**Keywords:** thalamopeduncular syndrome, cervical dystonia, torticollis, artery of Percheron, vascular dementia

## Abstract

Bilateral thalamic infarctions are usually caused by occlusion of the "Artery of Percheron" (AoP). Thalamopeduncular syndrome is among the most common presentations of AoP occlusion. A 59-year-old male presented abrupt decreased level of consciousness. After several weeks, on regaining consciousness, he exhibited oculomotor abnormalities, ataxic gait, cervical dystonia, and cognitive and behavioral changes. Magnetic resonance imaging disclosed thalamic, subthalamic, mammillary and midbrain infarction. Clinical features suggestive of bilateral thalamopeduncular syndrome were identified. Besides the presence of cognitive impairment and behavioral symptoms, cervical dystonia was evident, possibly resulting from interruption of the interconnections among basal ganglia, thalamus, subthalamus, midbrain and cerebellum.

## Abstract (pt)

Infartos talâmicos bilaterais são em geral ocasionados por oclusão da "Artéria de Percheron" (AdP). A síndrome talamopeduncular está entre as apresentações clínicas mais comuns da oclusão da AdP. Um homem de 59 anos apresentou rebaixamento abrupto do nível de consciência. Após algumas semanas, ao recobrar a consciência, apresentava anormalidades oculomotoras, marcha atáxica, distonia cervical e alterações cognitivas e comportamentais. A imagem por ressonância magnética evidenciou infartos talâmico, subtalâmico, mamilar e mesencefálico. O quadro clínico foi sugestivo de síndrome talamopeduncular. Além da presença de comprometimento cognitivo e transtornos de comportamento, estava presente distonia cervical, que pode resultar da interrupção das interconexões entre gânglios da base, tálamo, subtálamo, mesencéfalo e cerebelo.

## INTRODUCTION

The thalamus plays a crucial role in several distinct circuits associated with cognitive, behavioral, motor and sensory functions; hence vascular lesions of these structures may produce a heterogeneous range of clinical features.^1^ The structure is supplied by branches of the posterior cerebral artery and the posterior communicating artery, constituting vascular territories related to different syndromes. Among these, the thalamic paramedian territory, supplied by the paramedian arteries and their variations (branches of the P1 segment of the posterior cerebral artery, known collectively as the "artery of Percheron" - AOP), has received special attention.^2^ Thalamic infarcts associated with occlusion of this artery are considered rare, although epidemiological data from large population studies are not available.^1,2^

The aim of the present study was to report a case of bilateral paramedian thalamic infarct with mesencephalic extension (thalamopeduncular syndrome), in which the clinical features included cervical dystonia, eye movement abnormalities, ataxia, cognitive impairment and behavioral disorders. This study is a branch of a project on Vascular Cognitive Impairment, which was approved by the Ethics Committee of the Institute of Psychiatry, Federal University of Rio de Janeiro (CEP-IPUB-UFRJ), under protocol number 416.952. An informed consent form authorizing the use of his clinical data for research purposes was signed by the patient and a responsible proxy.

## CASE REPORT

A 59-year-old right-handed man, with 12 years of education, was admitted to the emergency unit in December 2008. At admission, his spouse reported that, earlier that day, he had presented abrupt drowsiness, followed by decreased level of consciousness, progressing to coma. There was no history of headache, fever or seizure related to the event. Past medical history included hypertension (with inadequate treatment compliance) and dyslipidemia, low exercise and smoking habits. No history of alcohol or substance-related disorders was identified through interview with the spouse. Laboratory tests were not suggestive of infections, nutritional deficits or electrolyte imbalance.

The patient regained consciousness after 3 weeks. At this time, important changes in the subject's behavior were identified through a structured interview with his wife (Neuropsychiatric Inventory – NPI). For instance, he had lost interest in his usual activities, such as singing, and he was less willing to engage in a conversation with his family and friends (these aspects were scored within the Apathy domain of the NPI). He had no feeling of sadness or guilt and scores on the Depression domain of the NPI were due to affirmative response to the question about loss of interest in leisure activities. Moreover, he claimed that a stranger was living in his house, although he could not see or hear what he/she said – that was scored as a Delusion symptom on the NPI.

Cognitive changes were assessed by a battery of neuropsychological tests (Table 1).

**Table 1:** Cognitive and behavioral evaluation: comparison with normative data.

	Case subject	Normative value	Comment
MMSE	23	26/30	bellow cut-off
CAMCOG (total)	74	90.20 (6.82)	below -2 sd
Orientation	10	9.57 (0.83)	-
Language	23	26.39 (1.93)	below -1.5 sd
Memory	16	23.10 (3.52)	below -2 sd
Attention	2	5.67 (1.36)	below -2.5 sd
Abstract thinking*	4	6.00 (1.77)	below -1 sd
Praxis	7	10.73 (1.20)	below -3 sd
Calculation	2	1.88 (0.32)	-
Visual perception	8	5.70 (1.60)	below -1 sd
Touch perception	2	1.93 (0.26)	-
VF (animals)*	7	13	below cut-off
CLOX-I*	9	10/15	-
CLOX-I*	11	12/15	-
TMT A*	248 sec	35.10 (10.94)	below -4 sd
TMT B*	incomplete (300 sec)	78.84 (19.09)	NA
PFAQ	9	2.35	deficient
CDR	1	0	mild VaD
HIS	12	>7	VaD
NPI	score		
Delusion	4		
Hallucination	4		
Depression	4		
Anxiety	4		
Apathy	9		
Irritability	2		
Total	27		

* Fronto-executive functions tasks; sd: standard deviation; NPI values: only for the items scored; MMSE: Mini-Mental State Examination; HIS: Hachinski Ischemic Score; PFAQ: Pfeffer’s Functional Activities Questionnaire; CDR: Clinical Dementia Rating; CLOX: Clock Drawing task; TMT: Trail Making Test; NPI: Neuropsychiatric Inventory.

**Figure 1:**
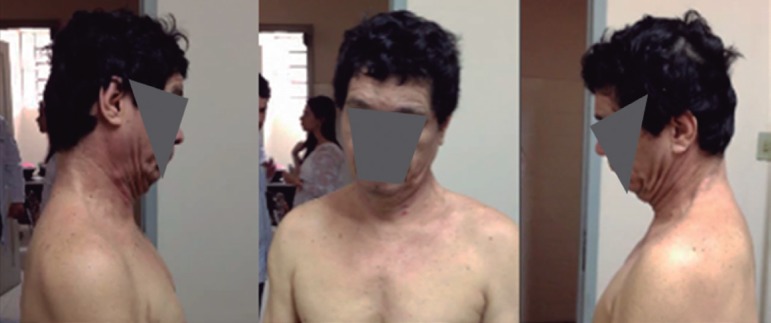
Cervical dystonia: dystonic head posture with anteroflexion.

Physical examination revealed the presence of cervical dystonia (spasmodic torticollis-anterocollis) (Figure 1), ataxic gait, vertical gaze palsy (upward and downward), convergence insufficiency, mydriatic nonreactive pupils, and light intolerance.

**Figure 2:**
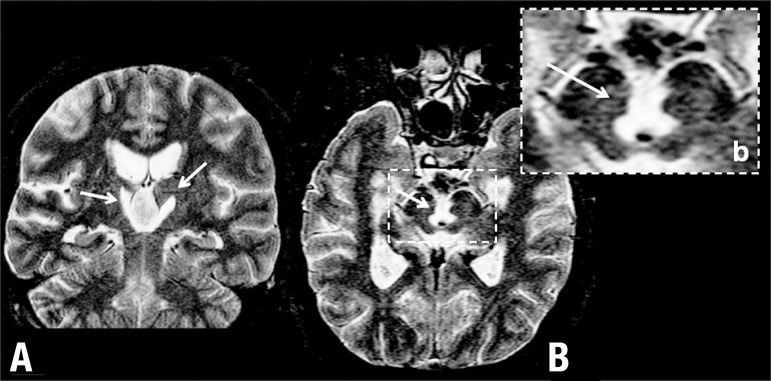
MR - T^2^ acquisition. [A] arrows indicating bilateral paramedian thalamic lesions. [B] arrow indicating mesencephalic midline lesion, [b] magnified inset of upper mesencephalic lesion (including approximately, from ventral to dorsal-ward, mainly the interpeduncular nucleus, ventral tegmental area, raphe nuclei, oculomotor nuclei, medial longitudinal fasciculus, ventral part of periaqueductal grey).

**Figure 3:**
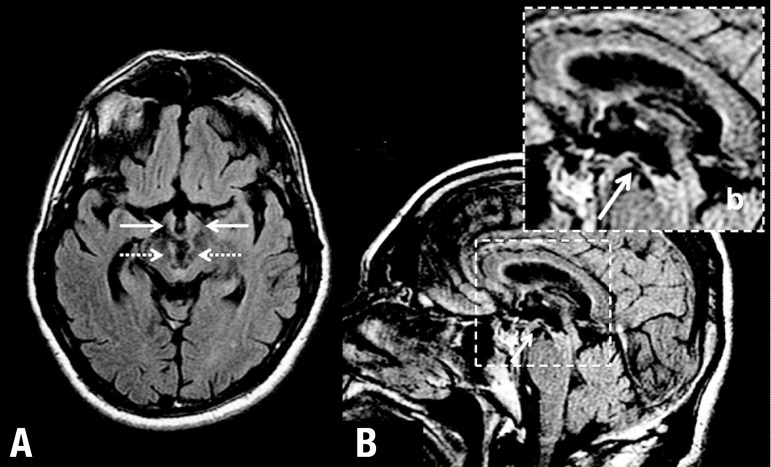
MR - FLAIR acquisition. [A] (axial section): solid arrows indicating basal hypothalamus: structural changes (including medial parts of the mammillary bodies), broken arrows indicating mesencephalic midline lesion. [B] (sagital section): arrow indicating basal hypothalamic lesion, continuing posteriorly with mesencephalic lesion, [b] inset displaying the area with magnified view.

**Figure 4:**
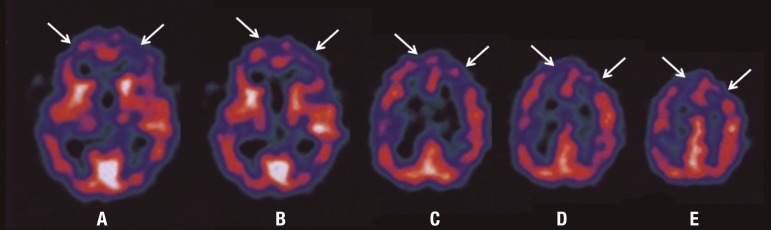
SPECT – axial. A to E: arrows indicating frontal hypoperfusion areas.

Structural MRI disclosed bilateral paramedian thalamic lesions, extending to the subthalamus, midline posteroventral hypothalamus (mammillary bodies – medial part), with right predominance in all these regions and also symmetrical lesions in the midline of the rostral mesencephalon, from the interpeduncular fossa to the anterior periaqueductal grey (Figure 2). MRI angiography and ultrasonography studies were not performed due to technical reasons. Figures 2 and 3 depict the structures showing damage in the case. SPECT revealed multiple cortical hypoperfusion areas, with predominance in bilateral dorsolateral and basal frontal lobes. Moreover, minor changes in the right temporoparietal and in the left parietal projections were identified (Figure 4).

Treatment strategies, besides general clinical measures, included the prescription of a cholinesterase inhibitor, without clear response, and injection of Botulinum toxin for the cervical dystonia, which promoted a partial response. Further follow-up was lost as the patient did not continue to attend the medical consultations a few months after the initial assessment.

## DISCUSSION

The patient reported in the present case developed clinical features suggestive of bilateral thalamopeduncular vascular syndrome - the classic triad of acute decrease in level of consciousness, cognitive impairment (mainly in memory and learning abilities), and vertical gaze abnormalities.^2-4^

Possible correlations between some of the clinical findings and the neuroimaging changes warrant discussion. The cognitive and behavioral findings, which can be recognized as a picture of vascular cognitive impairment in the presence of mild vascular dementia (Table), may be due to the bilateral paramedian thalamic and hypothalamic lesions, as well as the frontal changes seen on SPECT imaging. Frontal hypoperfusion, as shown by the SPECT, might be secondary to diaschisis, as a consequence of disruption of the connections between prefrontal cortex and the thalamus nuclei. The oculomotor abnormalities could be associated with the mesencephalic lesions, extending from the interpeduncular fossa to the anterior periaqueductal gray, based on the reasonable assumption that the upper segment of the medial longitudinal fasciculus and the pretectal nuclei (related to vertical gaze and convergence), as well as the pupillary nuclei (Edinger-Westphal's, associated with the photomotor reflex) were affected. The ataxic gait may be related to the lesion of the superior cerebellar peduncles decussation.^1,2^ Finally, cervical dystonia related to cerebrovascular disease, as appearing here, may be the result of disruption of intricate interconnections among basal ganglia, thalamus, subthalamus, midbrain and cerebellum.^5,6^ This is regarded as a rare condition by the authors, as seen in Lee and Marsden's report describing only one case with torticollis related to subthalamic lesion among other 62 subjects with focal lesion in the thalamus and/or subthalamic region.^6^ Other studies have suggested associations between dystonia and lesions in the putamen, caudate, pallidum, thalamus, rostral mesencephalon and cerebellum. Another hypothesis attributes cervical dystonia to a disorder in midbrain networks implicating the superior colliculi and the striato-nigro-collicular pathway; the latter associated with both ocular and cephalic motricity. Disruption in these circuits may lead to hyperexcitability of the premotor neurons, which may activate tecto-reticulospinal and tectospinal pathways and provoke stimulus to the motor neurons in the upper cervical spinal cord that could result in cervical dystonia.^5-7^ Thus, lesions in the thalamus, subthalamus and midbrain, as observed in the present case, might have contributed to the development of cervical dystonia through the interruption of various different structures.

Other aspects of the case are noteworthy. Lesions in the thalamic-subthalamic-mesencephalic regions might have impaired the subject's drive-motivation and sense of reality, which manifested as apathy and delusions. These changes in the patient's behavior persisted during the months following the stroke and did not show a fluctuating pattern, that could suggest the presence of a confusional state.^1,8^ This is consistent with the preserved orientation and adequate performance on the calculation task (depicted in Table).

Vascular risk factors (hypertension, dyslipidemia, smoking) and a sudden onset, without evidence of embolic source, may indicate that the occlusion of the AoP may be associated with an atherothrombotic mechanism as the probable etiology of the stroke. Furthermore, differential diagnosis of bilateral thalamic lesions might be challenging in some cases. However, the presence of vascular risk factors and the sudden onset, as well as the peculiar pattern of the brain insult, allowed exclusion of several conditions (for instance, metabolic processes and neoplasm)^9,10^ that can mimic the picture reported.

In conclusion, this report illustrates a case of thalamopeduncular syndrome, which besides cognitive impairment and behavioral disorders, presented an unusual motor feature, cervical dystonia, manifestations that could be clearly correlated with the different brain regions affected by the characteristic cerebrovascular lesion.
